# Special Issue “Metformin: Mechanism and Application 2023”

**DOI:** 10.3390/ph19081263

**Published:** 2026-08-11

**Authors:** Agnieszka Śliwińska

**Affiliations:** Department of Nucleic Acid Biochemistry, Medical University of Lodz, 92-213 Lodz, Poland; agnieszka.sliwinska@umed.lodz.pl

Metformin remains the cornerstone of pharmacological therapy for type 2 diabetes mellitus (T2DM). Originally derived from compounds identified in *Galega officinalis* L., this synthetic biguanide has been used in clinical practice for more than six decades. Although initially introduced as an antihyperglycemic agent, it is now recognized as a drug with diverse biological activities that extend well beyond glucose regulation. The articles gathered in this Special Issue reprint reflect the expanding scope of metformin research, encompassing its pharmacokinetics, molecular mechanisms of action, adverse effects, biomarker discovery, and emerging therapeutic applications in oncology, neurodegenerative diseases, ischemia/reperfusion injury, gestational diabetes mellitus, and complications associated with systemic therapies. Together, these studies illustrate how the perception of metformin has evolved from that of a glucose-lowering drug to a broader metabolic regulator with potential applications across multiple fields of medicine.

Froldi (Contribution 1) opens this Special Issue reprint with a comprehensive overview of the pharmacokinetic and pharmacodynamic properties of metformin. The review emphasizes the critical role of organic cation transporters in drug distribution and renal elimination and discusses the mitochondrial mechanisms responsible for its biological activity. In particular, inhibition of respiratory chain complex I increases the intracellular AMP/ATP ratio, leading to activation of AMP-activated protein kinase (AMPK), a key regulator of cellular energy homeostasis. This signaling pathway provides a mechanistic basis for many of the metabolic and cytoprotective effects attributed to metformin.

Advances in our understanding of these molecular mechanisms have also strengthened interest in repurposing metformin for indications beyond diabetes. Roberts et al. (Contribution 2) summarize evidence from a broad range of experimental models, highlighting the drug’s anti-inflammatory, antiviral, neuroprotective, and cardioprotective properties. Their review also discusses the growing evidence linking metformin to epigenetic regulation, further illustrating the complexity of its biological actions. Overall, the available data suggest that metformin exerts a considerably wider spectrum of effects than originally anticipated.

As an orally administered drug, metformin interacts extensively with the gastrointestinal tract, making this organ system central to both its therapeutic effects and its adverse reactions. Petakh et al. (Contribution 3) examined changes in the gut microbiome of patients with T2DM who developed COVID-19 and reported a substantial reduction in microbial alpha diversity. Antibiotic treatment further aggravated this dysbiosis, whereas metformin therapy was associated with partial restoration of microbial diversity. These observations suggest that metformin may counterbalance microbiome disturbances and potentially contribute to improved clinical outcomes in diabetic patients affected by COVID-19.

Gastrointestinal intolerance, however, remains one of the most common reasons for poor adherence to metformin therapy. To address this clinically important issue, Szymczak-Pajor et al. (Contribution 4) conducted a meta-analysis of 26 randomized controlled trials involving more than 41,000 participants. Their findings indicate that combining metformin with other oral glucose-lowering agents may increase the incidence of nausea and vomiting. In contrast, concomitant probiotic supplementation reduced the risk of metformin-associated diarrhea, bloating, and constipation, suggesting a practical approach to improving the long-term tolerability of treatment.

The broad biological activity of metformin is particularly evident in its capacity to protect tissues against both acute and chronic injury. Among the most extensively investigated areas is cardiovascular disease. Osorio-Llanes et al. (Contribution 5) review the molecular mechanisms underlying metformin-mediated protection during myocardial ischemia/reperfusion (I/R) injury, highlighting evidence that the drug limits cardiomyocyte death and attenuates left ventricular dysfunction. These effects appear to result from coordinated regulation of cellular energy metabolism, apoptosis, autophagy, and mitochondrial oxidative stress. By integrating findings from experimental studies, the authors demonstrate how metabolic modulation may ultimately improve cardiac resilience following ischemic injury.

Similar mechanisms have been implicated in neurodegenerative disorders. Isop et al. (Contribution 6) summarize current evidence regarding the potential role of metformin in Alzheimer’s and Parkinson’s diseases, with particular emphasis on the LKB1–AMPK signaling pathway. Beyond its metabolic actions, metformin has been reported to exert antioxidant and immunomodulatory effects that may contribute to neuronal protection. At the same time, the authors acknowledge an important limitation of the available evidence. While preclinical studies have consistently demonstrated encouraging neuroprotective effects, comparable benefits have yet to be confirmed in well-designed clinical trials. Further clinical investigation will therefore be essential to determine whether these experimental observations can be translated into meaningful therapeutic outcomes.

Another area in which metformin has attracted considerable attention is oncology. Corleto et al. (Contribution 7) provide a comprehensive overview of research exploring its potential role in breast cancer prevention and treatment, following the evolution of evidence from experimental models to clinical studies. Although numerous investigations have reported antitumor effects, the clinical data remain inconsistent. The authors attribute this variability to several factors, including differences in treatment regimens, metabolic status, menopausal status, dietary habits, and tumor subtype. Their review emphasizes that future studies should adopt a more personalized approach when evaluating metformin as an adjunct to anticancer therapy.

The heterogeneity of clinical responses observed across both metabolic and oncological settings has stimulated increasing interest in identifying biomarkers that may predict treatment efficacy. Addressing this issue, Mujammami et al. (Contribution 8) applied an untargeted serum lipidomics approach to characterize metabolic changes associated with obesity, T2DM, and metformin therapy. Their analysis identified 54 lipid species that were significantly altered following metformin treatment in obese individuals with diabetes, including triacylglycerols, plasmenyl phosphatidylcholines, phosphatidylglycerols, sterol lipids, and mannosyl-phosphoinositol ceramides. These findings highlight the potential value of lipidomic profiling for monitoring treatment response and provide additional insight into the systemic metabolic effects of metformin.

A complete evaluation of metformin’s therapeutic potential also requires consideration of the situations in which its beneficial effects may be limited. The final contributions included in this Special Issue address several clinically relevant areas where the response to metformin appears to depend on specific disease-related factors, patient characteristics, or treatment context.

The use of metformin during pregnancy remains one of the most debated areas of its clinical application. Tocci et al. (Contribution 9) discuss the current challenges associated with metformin therapy in gestational diabetes mellitus (GDM), a condition in which lifestyle modification and insulin continue to represent the standard approaches. Although metformin has emerged as a potential alternative for selected patients, its ability to cross the placenta remains an important consideration. Available evidence suggests an acceptable short-term safety profile for the mother; however, the long-term metabolic and developmental consequences of fetal exposure have not yet been fully established. As a result, the optimal role of metformin in GDM management continues to be actively investigated.

The effectiveness of metformin may also be influenced by the presence of coexisting endocrine disorders. In a study involving postmenopausal women, Krysiak et al. (Contribution 10) demonstrated that euthyroid autoimmune thyroiditis (Hashimoto’s disease) significantly reduced the gonadotropin-lowering effects of metformin. The extent of the reduction in follicle-stimulating hormone (FSH) and luteinizing hormone (LH) was associated with thyroid antibody concentrations and baseline insulin sensitivity, suggesting that chronic autoimmune inflammation may interfere with some endocrine actions of the drug. These findings further emphasize the importance of considering the broader metabolic and inflammatory environment when evaluating treatment responses.

The limitations of metformin in specific pathological conditions are further highlighted by experimental evidence from toxicological models. Alhowail and Aldubayan (Contribution 11) investigated whether metformin could protect against hypothyroidism and cardiotoxicity induced by CMF chemotherapy (cyclophosphamide, methotrexate, and 5-fluorouracil) in male rats. Despite the recognized antioxidant and cardioprotective properties of metformin, co-treatment failed to prevent the CMF-associated reduction in thyroid hormone levels (T3 and T4) or the increase in cardiac injury markers, including troponin I, creatine kinase (CK), and CK-MB. These results demonstrate that the protective effects of metformin are not universal and may vary depending on the underlying mechanism of tissue injury.

Taken together, the studies presented in this Special Issue reprint reinforce the concept that metformin represents far more than a conventional antidiabetic medication. Its influence on the gut microbiota, lipid metabolism, cellular stress responses, ischemic injury, neurodegenerative pathways, and cancer biology highlights the remarkable breadth of its pharmacological activity. At the same time, the evidence summarized here emphasizes that the clinical effects of metformin are shaped by multiple interacting factors, including sex, comorbidities, inflammatory status, disease characteristics, and individual patient variability. Future progress will depend on integrating mechanistic insights with carefully designed clinical studies to better define where metformin provides meaningful benefit and which patient populations are most likely to respond favorably.

